# Hôtel Dieu

**Published:** 1832-07

**Authors:** 


### HÔTEL DIEU.

*Diagnosis; Dislocations of the Hip.* (*From "Selections from the Clinical Lectures delivered at the Hôtel-Dieu in Paris, by* Baron Dupuytren. *London Med. and Surg. Journal.*)

The diagnostic of fractures of the neck of the thigh bone is not without great difficulty, and the best surgeons often arrive at an uncertain judgment. There are some persons who present all the rational signs of this injury without its presence; and others, on the contrary, who offer no sign when it really exists. A fall on the hip will contuse the muscles and the articulation, and simulate this fracture; and though the same cause may produce fracture, the person rises and walks. Even persons have had the neck fractured, and regained the use of the limb, without any shortening, and the fragments have not been displaced for two, three, five, ten, or thirty days, and then by some movement of the patient, during which time the nature of the injury was unknown. What is the cause of this peculiarity which authors have signalized, and which I myself have often observed, and of which Sabatier makes mention in the Memoirs of the Academy of Surgery, in which he has cited several examples? It arises from this, that the fracture has occurred in the articular capsule, the fragments rest in their place, the inferior pressing upon the superior. But it may be asked, how is it that, after such a length of time, the displacement occurs, and the fracture becomes evident? It is because the relative position of the fragments change, either by the weight of the body, or by muscular action, or by the wearing of some of the fragments. The shortening and deviation of the limb, which supervene on the displacement of the fragments, leave no doubt of the existence of fracture, if there has been no other cause but a fall some days before. The two preceding signs do not manifest themselves sometimes sooner than after fifty, sixty, or eighty days of treatment by extension and repose, and are then known by the yielding of the callus to the contraction of the muscles or weight of the body.

Before I proceed further, I shall say a few words on the causes of this consecutive displacement.

It is known that in the early period of the formation of the callus of the body of the long bones, the union often yields and produces deformities in fractures which were perfectly adjusted, and when a cure was hoped for, without any accident. Who has not seen in oblique fractures of the femur, so difficult to be maintained with exactitude, that the callus yields to the weight of the limb, and deforms it when the patient attempts to walk, at a time which appears too distant for the slightest apprehension?

This is what exactly happens in fractures of the neck of the thigh bone, in which there has been no displacement at the early period of the injury, or when the displacement was slight at first. At the end of two or three months, the provisionary callus yields to the weight of the limb on which the patient supports himself, and the fragments cease to be in apposition, a deformity takes place, and a shortening is produced. I have seen those shortenings take place at the end of two, three, four months, and even later. From the observation of these facts there results an important indication, to which I shall direct your special attention, that it is necessary to keep patients in the apparatus destined for fractures of the neck of the thigh bone, during 100, 120, 140 days, or even more, so as to give the callus time to acquire sufficient solidity.

The weight of the limb, but above all that of the body on the broken member, ought to be regarded as the great cause of displacement, whether primitive or consecutive, carrying the superior fragment inferiorly, and the inferior superiorly. But an active power of displacement consists in the prolongation of the action of the cause which occasioned the fracture, the same cause produces the most remarkable effect. When it is carried too far, it forces the superior fragment into the spongy tissue of the upper extremity of the inferior fragment, and consolidation rapidly takes place in this situation. There will be deformity, shortening, and permanent deviation of the inferior member, and in a direction which will vary according to the point at which this anormal union occurs. It may happen before or behind. Here the shortening of the inferior member takes place in the spongy substance of the inferior fragment.

Many pieces of pathological anatomy are preserved at the Museum of the Hôtel Dieu, and represent fragments consolidated in this manner; they have been exhibited in this amphitheatre, and have convinced every one of the reality of this interesting fact. It is useful to note this cause of deviation; it accounts for some exceptional facts of deviation of the foot inwards, in fracture of the neck of the femur; exceptional facts, which have been observed by many authors.

In fine, there exists another, and the last cause of displacement of the fragments in fracture of the neck of the thigh-bone; a cause incessantly acting, and against which the surgeon always directs his efforts, to prevent vicious consolidations and deformities; I mean muscular action. It is this which explains that displacement most readily occurs when the callus is not as yet formed. This consideration very properly sanctions the division of the symptoms of fracture of the neck of the thigh-bone into primitive and consecutive, according as the shortening occurs before or after the formation of the callus.

The primitive symptoms exist when, in a fall on the heel or knee, the shortening and deviation occur at the instant; in this case the superior fragment rests in its place, and the inferior ascends; but a vertical fall is the rarest cause of the fracture of the neck of the femur; and in falls on the great trochanter, which are commonest, the limb is elongated. There exists another cause of shortening, which until our time, was little known, and badly indicated. We attribute it to the action of the great muscles, the middle and small glutæi; but by rotation outwards these muscles are put in a state of relaxation, which is so with those which cause the deviation. The muscles of the legs and hams are considered as favoring this displacement, but they have no more influence than the others; for the relaxation, as that of the glutæi, is determined by rotation outwards. When the limb is placed on an inclined plane, the glutæi muscles are extended, and, nevertheless, the deviation ceases naturally. But it is to the adductor muscles, attached to the pubis and ischium, are due the deviation and shortening. These two symptoms appear when the patient makes muscular efforts to rise, or they follow a slight tonic contraction of these muscles, which do not give much resistance in the inferior fragment. It is this that causes the shortening after the formation of the callus, when the patient executes movements to rise up or walk. The action of the muscles, and the weight of the body, are then the real causes.

Let us pass to the enumeration and the appreciation of the symptoms, which will furnish us with important indications.

When there is displacement the fracture is easily recognized, but when there is none it may be suspected, without, nevertheless, our being able to decide with certainty. I suppose that the symptoms are well characterised, that there is shortening, deviation of the limb outwards, impossibility of rising from the bed. It is still necessary to ascertain if the limb preserves its shortening, or if it is destroyed by extension; if the great trochanter revolves on the axis of the femur, or on the extremity of the lever.

If the shortening is only a few lines, it is difficult to distinguish it from that produced by an accensional movement of the pelvis, the result of contusion; the diagnostic becomes more evident if it is of half an inch or an inch, &c.

Persons have attributed an old shortening to a recent fall, in order to induce the surgeon to attempt a cure; but an attentive examination will invariably enable him to arrive at the truth. When the displacement is not of long standing, but recent, it may be produced by luxation of the femur, or an ascension of the pelvis: here we must be cautious not to be imposed upon.

In the luxation forwards, the head of the femur passes on the horizontal branch of the pubis, elevating or lacerating the vessels or nerves; then there is shortening, but there exists before the pubis a hard tumor, which moves on rotating the femur. In the luxation in the sub-pubic, or ovalar region, the limb is still turned outwards, but is elongated, and here there is a thickening, resistance, and unaccustomed tension of the muscles; the hip is depressed, but projecting in fracture.

In the luxation above and outwards, the head of the femur is in the external iliac fossa, the limb is shortened, but the foot and patella are turned inwards, and the heel and the ham outwards.

It is true that in some cases of fracture the limb is turned inwards. Ambrose Paré, who was the first that described this disease, reports that he was sent for to a lady whose limb was short, and the great trochanter on the os ilium; he believed it was a case of luxation of the femur. He made efforts to reduce it, and applied a bandage; but after some days violent pains came on, the limb shortened itself again, and he found that the foot was turned inwards. John Louis Petit cites a similar case in his work on Diseases of the Bones. Bichat stated that Dessault considered this variety very common. I have only seen one case or two, and I think Bichat must have committed an error. But as this deviation inwards has been observed, what are the means of distinguishing fracture from luxation upwards and outwards? In the luxation, the round head of the femur is in the external iliac fossa. In fracture we can readily rotate the leg; in the former our efforts will be useless. In the luxation, we cannot elongate the limb without reduction and great efforts; but once reduced, there will be no farther displacement. In the fracture slight efforts will elongate the limb to its normal state, but displacement will immediately occur as soon as we suspend our efforts. It is therefore impossible to confound the two cases.

Lastly, there is a luxation downwards and backwards, which I have observed two or three times only; the limb is turned inwards, is sometimes a little lengthened; it cannot be brought to its natural position, except by efforts at reduction, and once reduced, the displacement is not produced again.

The grand distinctive character consists in the following difference: the shortening produced by a fracture yields to a slight effort, but that produced by dislocation is removed with difficulty, but when once removed it does not return without great force, such as that which first produced it, being used.

Disease of the hip-joint may produce shortening of the limb, but this cause is easily discovered. Conscripts simulate shortening and luxation of the limb, by walking on the point of the foot and elevating the other hip: the foot is turned outwards in fracture.

But it often happens that shortening will supervene after contusion of the hip, where there is no attempt at imposition. In such cases place the patient on a table, and apply a rule across from one anterior superior spine of the ilium to the other. If the shortening is produced by pain and contusion, the anterior spine of the affected side will raise itself above the other; elevate the depressed hip, and depress the other, and all shortening will disappear.

Let us now examine the deviation in which the point of the foot and patella are turned outwards, and the heel and ham inwards. When a person lies on the back, there is a distance of eight or ten inches between the toes of each foot; but in fracture the foot lies on its side, the heel is turned towards the opposite ankle, and the ham towards the knee. The contrary deviation of the foot inwards is of rare occurrence; it may be reckoned 1 in 100.

We have already explained the mode in which the deviation outwards is accomplished; but if the action of the adductors explain it well, it is also right to say there is another cause of deviation inwards, the obliquity of the fragments. If the body of the femur is fractured obliquely from below upwards and from before backwards, the point of the inferior fragment will be carried backwards, that of the superior before, and vice versa: but, in the fracture of the neck, if the internal fragment is carried backwards and the external forwards, there will be then deviation outwards; if, on the contrary, the fracture is oblique in the inverse sense, the deviation will be inwards; it is then by the obliquity of the fragment that these varieties of deviation can be appreciated.

After having spoken to you of the symptoms, it is natural to apprise you of the results of fractures abandoned to themselves; and finally to explain the treatment. I have constantly seen patients affected with fracture of the neck, who did not fail to present shortening of one, two, three, or four inches, and the deviation of the foot outwards, which rendered progression difficult; the great trochanter was elevated, approached the crest, and was directed backwards. But that which is especially to be known is, that a false joint is formed in the external iliac fossa.

Let us now inquire what are the material effects of fractures on the bones, commencing by the cotyloid cavity or acetabulum. I have two or three times observed the cavity fractured by the head of the femur: this accident was produced by a fall on the feet or knees. In such cases the head of the femur props the bottom of the cotyloid cavity, and as it offers more strength than this, it breaks it. The most remarkable case of this kind which I have observed was this; the bottom of the cotyloid cavity was fractured, and the injured head of the femur was passed entire into the pelvis; the neck, which was unbroken, was so forcibly engaged in this opening, that it was extremely difficnlt to withdraw it and reduce this new species of luxation. At an early age, the effort exercised on the bottom of the acetabulum can disunite the pieces of which the coxal bone (os innominatum) is composed, the point of union of which corresponds to the centre of the cavity.

In other cases the cavity is fractured without the head of the bone being displaced; but the most ordinary effects of fracture are perceived at the superior extremity of the femur. One of the first effects is a stellated fracture of the head of the femur, the neck remaining untouched. This case is very common in falls on the great trochanter, or on the soles of the feet, but more so when direct injuries are inflicted, as by fire-arms. I have had occasion to see a dozen cases in the month of July. I have remarked that it has often happened that the head of the femur having broken in pieces, the neck remained entire; there was no displacement of the fragments, no shortening of the limb, no deformity. This fracture may be mistaken for violent contusion of the articulation, and treated by general and local bleedings; in general it is unknown; the patients recover without deformity, and it is only when death arrives that its real nature can be determined.

The neck is oftener the seat of fracture, because it forms a lever; its thinness in the middle also favors a solution of continuity; sometimes it is broken near the head of the bone; the more ordinary fractures take place in the weakest part of the bone. This fracture may occur from below upwards, or from above downwards; at other times it occurs anteriorly or posteriorly; this depends upon the manner in which the fall takes place, the body being more or less inclined, or in an inverse sense. But the commonest fracture is seen at the base of the neck, as this results from falls on the great trochanter, which I have observed to be partial or complete, the neck being entirely separated from the body of the bone. When the trochanter resists the effect of the fall, that which most commonly occurs is a fracture of part of this eminence directed towards the small trochanter, and appears to be more at the superior part of the body than at the neck of the bone. Sometimes the fracture proceeds from the less to the great trochanter, or from the great, to the neck of the bone.

